# Supplementary figures and images for: Do aqueous solutions contain net charge?
Source: PLoS One. 2022 Oct 27;17(10):e0275953. doi: 10.1371/journal.pone.0275953 (PMC9612516; doi:10.1371/journal.pone.0275953)

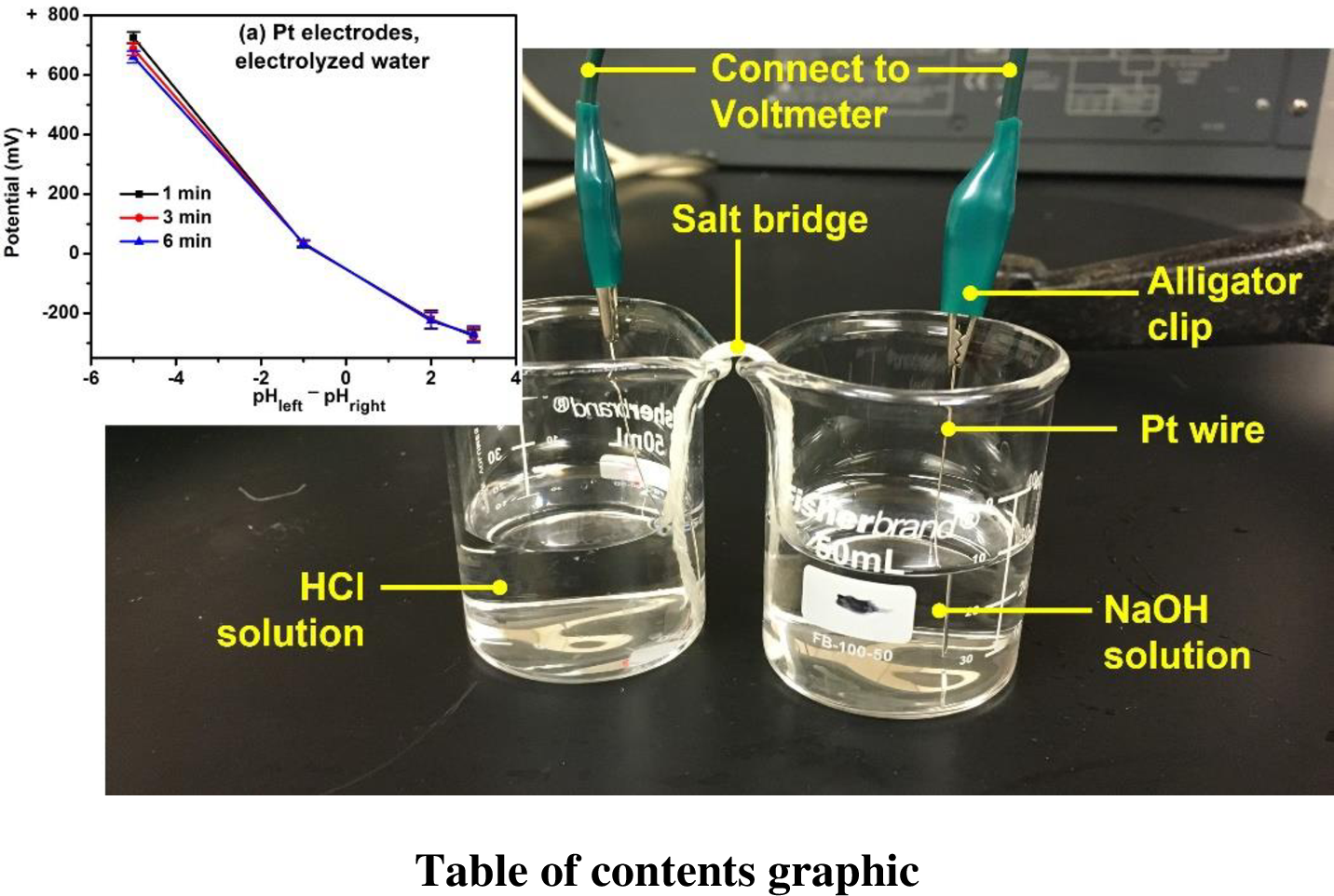

Supplement: S1 Graphical abstract — (TIF) [file pone.0275953.s001.tif]
