# Supplementary material for: Do aqueous solutions contain net charge?
Source: PLoS One. 2022 Oct 27;17(10):e0275953. doi: 10.1371/journal.pone.0275953 (PMC9612516; doi:10.1371/journal.pone.0275953)
Supplement: Attachment — Submitted filename: Response to Reviewers.docx [file pone.0275953.s002.docx]

**Response to Reviewers:**

| **Do aqueous solutions contain net charge?** | |
| --- | --- |
| **Reviewer 1** | |
| 1. The title of the manuscript gives an impression that we are asking the question to the Google engine. Isn’t it possible to change the title with more appealing pattern? | We thank the reviewer for this suggestion. We agree with the reviewer that this is a general question that people may search answers using Google. This is the reason why we believe this question is very important. We specifically discussed this situation in the first paragraph in Introduction. Therefore, we believe this simple and straightforward title may be a good choice. |
| 2. In Abstract at “..solutions that were more acidic were more positively charged, while solutions that were more basic were more negatively charged”, my suggestion to change it with “acidic solutions were positively charged while basic solutions were negatively charged”. | We agree with the reviewer’s suggestion. The sentence was changed in the Abstract.  “We found much the same when standard acids and bases were compared to reference solutions: acidic solutions were positively charged while basic solutions were negatively charged.” |
| 3. Normally we use the salt solution having the cations and anions which have similar mobility in solution but different from the ions present in our test solutions. But in this manuscript, sodium chloride (NaCl), potassium chloride (KCl), sodium sulfate  (Na_2_SO_4_), and potassium sulfate (K_2_SO_4_) were used while as the salt bridge cotton string soaked with saturated KCl solution  was used. If my belief is wrong, I want to see the logic behind it. | Thank you for the reminder.  We partly agree with the reviewer. We tried to Google what salt bridge solution we should use before we conducted the experiments. We found KCl very common (<https://en.wikipedia.org/wiki/Salt_bridge>).  We probably all know that the insertion of a concentrated KCl aqueous solution between two dilute aqueous electrolyte solutions can effectively eliminate the liquid junction potential (LJP), but there has virtually been no choice but a concentrated aqueous potassium chloride solution to minimize the LJP between two aqueous electrolyte solutions of different compositions.^1^  “Normally we use the salt solution having the cations and anions that have similar mobility in solution”. We are testing four: NaCl, KCl, Na_2_SO_4_, and K_2_SO_4_ solutions, hence, KCl should be an ideal salt solution because it has similar mobility to these solutions.  On-going research in our lab will consider using other salts as suggested by the reviewers.  The following sentence is added in the manuscript.  “The insertion of a concentrated KCl aqueous solution between two dilute aqueous electrolyte solutions can effectively eliminate the liquid junction potential. Therefore, salt bridges were all soaked in saturated KCl before use.” |
| 4. Regarding the data presented in Tables 1, 2: Just check the Table 1 (a) of electrical potential differences between acids (HCl) with differing pH values at different times to test stability. In cases of pH values 2 and 4, the potentials were found increased with increasing time from 1 min. to 6 min. while in cases of pH values 12 and 14 the potentials were found in  decreasing order with time elapsed (though not sharply in case of pH 14). Such type of order was not obeyed in case of the solution having pH 10.  In another case the data shown in Table 1 (b) regarding the electrical potential differences between acids (HNO_3_) and neutral solutions at different times to test stability. The potentials were found decreased with increasing time from 1 min. to 6 min. in cases of the solutions with pH values 2, 4, 12 and 14 but why not the trend was followed in case of the solution having pH 10? | We thank the reviewer for the comment.  We noticed it as well that in the pH 10 tests (Tables 1 & 2), the potentials registered fluctuated and the difference from 1-6 min was not as significant as other pHs that were tested.  The phenomenon is very interesting. We are not sure what is the exact reason behind it. It can be that the ionic strength or pH difference between the left beaker solution (positive terminal, pH 10, NaOH) and the right beaker solution (negative terminal, 1 µM NaCl) was not significant. Or as suggested in previous comment, it can be the salt bridge solution.  But the authors would like to mention that the potential registered may eventually stabilize, although it may take a long time.  Further research will be done in our on-going project.  The following sentences were added in the manuscript.  “We noticed that in the pH 10 tests (Tables 1 & 2), the potentials registered fluctuated and the difference from 1-6 min was not as significant as other pHs that were tested. We are not sure what is the exact reason behind it. It can be that the ionic strength or pH difference between the left beaker solution (positive terminal, pH 10, NaOH) and the right beaker solution (negative terminal, 1 µM NaCl) was not significant.” |
| 5. The results regarding the salt solutions of NaCl, KCl, Na_2_SO_4_ and K_2_SO_4_. Authors have mentioned that all these salt solutions at high concentrations had negative electrical potentials. The first logic gave for such controversial result as “the  periodic table may be incomplete, and that some elements may themselves bear charge”. This logic is much more controversial than the result mentioned. My suggestion to Authors that prior to write such paper why don’t they redefine the Periodic Table and publish their logic? | Plans are actually underway for one of the authors (GP) to write a book on the subject. Although the book draft is nearly complete, we expect, from experience, two to three years before it is finally published. Evidence will be presented to show that not all entries in the periodic table are necessarily correct, and that some elements may actually bear charge. Here, we merely allude to that possibility. The current work seems to us to be important enough that we hesitate to wait several years before it can be published. |
| 6. I suggest the authors to see the book: Bickmore B.R., Wander M.C.F. (2018) Aqueous Solutions. In: White W.M. (eds)  Encyclopedia of Geochemistry. Encyclopedia of Earth Sciences Series. Springer, Cham. https://doi.org/10.1007/978-3-319-  39312-4_3 | We thank the reviewer for the suggestion.  We also thank the reviewer for the time and effort on reviewing the manuscript. |
| **Reviewer 2** | |
| After going through the manuscript I found it to interesting but the authors reported only some data of their  experimental work and based on these data they claimed the title of the manuscript to taken as granted scientifically, which according to me will not be scientifically justified right now. The work is premature and needs more extensive experimental  and theoretical basis before the titled remark becomes scientifically established. | We thank the reviewer for the time and effort on reviewing the manuscript. Yes, from experience, we do understand that gaining community acceptance will take time.  Nevertheless, we believe we have included all the data necessary to draw the conclusions that we draw.  As mentioned in the first paragraph of the Introduction, “does a high pH value imply net negative charge?” or “does a low pH value  imply net positive charge?” are questions that consistently puzzle people. We believe our manuscript answers these questions scientifically, although we agree that it will take time for the scientific community to evaluate, and hopefully accept our conclusions. |

1.	Takashi, K.; Takahiro, Y., A New Salt Bridge Based on the Hydrophobic Room-Temperature Molten Salt. *Bulletin of the Chemical Society of Japan* **2006,** *79*, (7), 1017-1024.
