# Supplementary material for: Do aqueous solutions contain net charge?
Source: PLoS One. 2022 Oct 27;17(10):e0275953. doi: 10.1371/journal.pone.0275953 (PMC9612516; doi:10.1371/journal.pone.0275953)
Supplement: Attachment — Submitted filename: Response to Reviewers.docx [file pone.0275953.s003.docx]

**Rebuttal letter:**

After we addressed reviewer comments from the first review, our manuscript was rejected by the academic editor based on subsequent comments from one reviewer. Our responses are given below. We do not believe any further manuscript revision is necessary. Thank you for your consideration.

| **Do aqueous solutions contain net charge?** | |
| --- | --- |
| **Academic Editor** | |
| Thank you for submitting your manuscript to PLOS ONE. After careful consideration, we have decided that your manuscript does not meet our criteria for publication and must therefore be rejected.  Specifically, I am sorry that we cannot be more positive on this occasion, but hope that you appreciate the reasons for this decision. | We were disappointed to receive the rejection notice because the revisions required by the reviewers were relatively minor and we addressed them carefully and respectively.  We have put long efforts into this manuscript to make the rationale clear to the reader and to support our conclusions. The Academic Editor made a decision based on the one reviewer whose comments led us to conclude bias.  Dr. Gerald Pollack is not only a senior author on this manuscript, but also Editor-in-Chief of the Journal WATER <https://waterjournal.org/>. This is his response to the Academic Editor regarding that one negative review:  “Frankly, if a review of this kind were to come to my desk as Editor-in-Chief of our journal, I would not only discount it, but also consider whether to invite him/her for another manuscript review in the future. I would be embarrassed to return such a review to an author.  We therefore wonder: One reviewer was satisfied with our presentation and our logic. This second reviewer poses arguments that strike us as hollow. Is there anything we can do to make this manuscript acceptable?”  We understand that according to the policies of PLOS ONE, authors are reasonably permitted to draw their own conclusions, so long as the experiments were soundly performed. <https://journals.plos.org/plosone/s/editorial-and-publishing-policies> We have done so. The critical reviewer appears to offer no substantive criticism of our experiments or analysis. |
| **Reviewer 1** | |
| Regarding the manuscript which I reviewed earlier and then you sent to me as the revised version, I still have many objections some of which are… | The reviewer provided suggestions in the first review round, which we addressed. It seems those revisions were for the large part ignored, or discounted, without reason.  The reviewer now says he/she still has many objections, but no detail is provided. Hence, we feel the rejection comes without any solid reason.  For a young researcher like myself, such an attitude is discouraging. |
| 1. Regarding the quarry of title: Though not satisfied with the logic of Author, it doesn't make any sense to deny for publication on such basis. | The reviewer doesn’t agree with the logic. However, no reason is presented as to WHY he disagrees with the logic. What meaning can one assign to such an empty comment? |
| 2. Correction done in accordance with my suggestion. | Yes, we made the modest changes suggested by the reviewer. We appreciate the acknowledgement. |
| 3. Regarding the use of salt solution in salt bridge: I am not satisfied at all. Furthermore writing "On-going research in our lab will consider using other salts as suggested by the reviewers." can't be taken as the positive token to consider the present manuscript. | Salt solution with 3M KCl: This is the standard salt-bridge solution used routinely for practically a century. No reason has been given as to why it might be inadequate. Why should we be held to a standard beyond that of so many other authors? |
| 4. Regarding electrical potential difference: I am not satisfied with the explanation as "Further research will be done in our on-going project.” | Naturally, more work can be done in the future. It always can. And, it should. The question is whether we have done enough to make our point. The reviewer gives no reason why he/she thinks we have not. |
| 5. Regarding controversial statement related with Periodic Table: The author has mentioned as "Plans are actually underway for one of the authors (GP) to write a book on the subject. Although the book draft is nearly complete, we expect, from experience, two to three years before it is finally published." On this ground if we accept author's logic right now, it can be disaster. | Finally, when the reviewer comments on a forthcoming book that he has not seen, and states: “if we accept the author’s logic right now, it can be a disaster,” what can we possibly think? Why would a reviewer be fearful of a book he has never seen? And, what can we conclude about the reviewer’s objectivity.  --  In sum, we can identify no *substantive* reason why the reviewer rejects our manuscript. |
