# Supplementary material for: Do aqueous solutions contain net charge?
Source: PLoS One. 2022 Oct 27;17(10):e0275953. doi: 10.1371/journal.pone.0275953 (PMC9612516; doi:10.1371/journal.pone.0275953)
Supplement: Attachment — Submitted filename: Response to Reviewers.docx [file pone.0275953.s004.docx]

Dear Editor and Reviewer,

Thank you for taking the time and effort to review our manuscript.

We are happy to remove the offending sentence, and have done so.

Regarding the other comment, about the proposed title revision, we felt the newly suggested title was unclear. We asked the opinion of several colleagues, and everyone came back with similar comment. In the proposed new title, “Do aqueous solutions contain a semi-net charge?”, every one of the people we asked responded similarly: unclear. The offending issue: what is “semi-net charge”? All felt that the term was vague. We feel the same.

While we do not wish for the title argument to get in the way of publication, at the same time, we would not feel comfortable with the proposed title revision. Therefore, if the reviewer is willing to come up with an alternative title that is as clear and direct as our original, we would be happy to consider it.

Regarding the related comment, that we’ve produced “no any direct data of net charge,” the reviewer is correct. However, **no direct measurement of charge is possible.** In the electrical engineering education of one of the manuscript’s authors (GP), it became clear that the way of assessing charge is through measurement of potential difference. That is the accepted method. Everyone uses it. So, while the reviewer is correct in asserting that we’ve not actually *measured* net charge, net charge is commonly inferred from measurements of potential difference. We doubt that any physicist would disagree. That is what we’ve done. We hope it can be accepted by the reviewer.

We therefore hope you are willing to reconsider. We have modified the manuscript with regard to the one comment on sentence deletion, but hope you consider our argument as to why we feel that the comment on change of title using “semi-net charge” is unwarranted.

Best wishes,

Tao Ye, PhD, Assistant Professor

Gerald Pollack, PhD, Professor
